# Supplementary material for: The causal role of circulating inflammatory markers in osteoporosis: a bidirectional Mendelian randomized study
Source: Front Immunol. 2024 Jul 18;15:1412298. doi: 10.3389/fimmu.2024.1412298 (PMC11291241; doi:10.3389/fimmu.2024.1412298)
Supplement: Supplementary file 1 [file DataSheet_1.zip › supplementary material/CIM-OSTEOPOROSIS /GCST90274785.csv_forest.pdf]

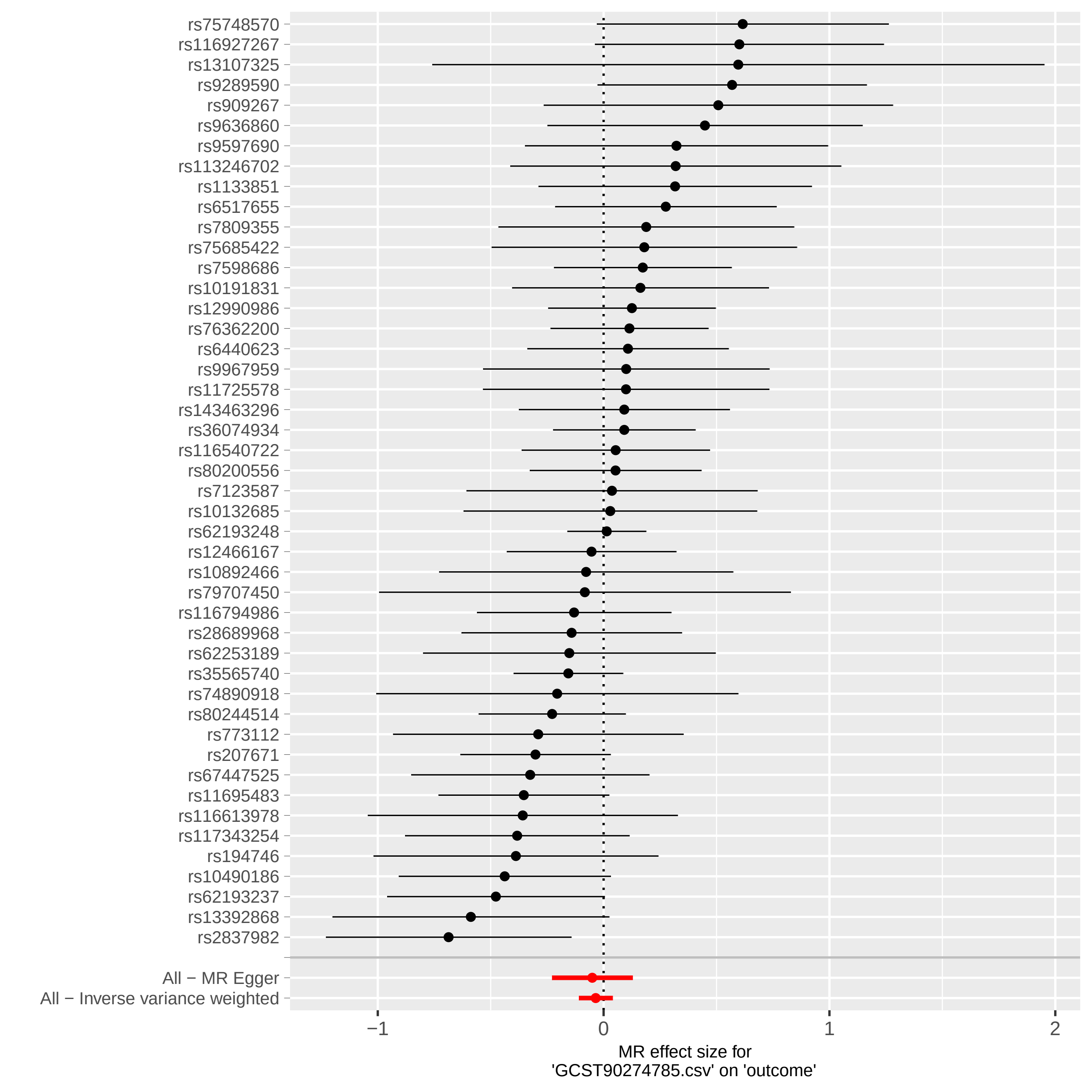
rs75748570
rs116927267
rs13107325
rs9289590
rs909267
rs9636860
rs9597690
rs113246702
rs1133851
rs6517655
rs7809355
rs75685422
rs7598686
rs10191831
rs12990986
rs76362200
rs6440623
rs9967959
rs11725578
rs143463296
rs36074934
rs116540722
rs80200556
rs7123587
rs10132685
rs62193248
rs12466167
rs10892466
rs79707450
rs116794986
rs28689968
rs62253189
rs35565740
rs74890918
rs80244514
rs773112
rs207671
rs67447525
rs11695483
rs116613978
rs117343254
rs194746
rs10490186
rs62193237
rs13392868
rs2837982
All − MR Egger
All − Inverse variance weighted
−1
0
1
2
MR effect size for
'GCST90274785.csv' on 'outcome'
